# Computation of significance scores of unweighted Gene Set Enrichment Analyses

**DOI:** 10.1186/1471-2105-8-290

**Published:** 2007-08-06

**Authors:** Andreas Keller, Christina Backes, Hans-Peter Lenhof

**Affiliations:** 1 Center for Bioinformatics, Saarland University, Building E1 1, 66804 Saarbrücken, Germany

## Abstract

**Background:**

Gene Set Enrichment Analysis (GSEA) is a computational method for the statistical evaluation of sorted lists of genes or proteins. Originally GSEA was developed for interpreting microarray gene expression data, but it can be applied to any sorted list of genes. Given the gene list and an arbitrary biological category, GSEA evaluates whether the genes of the considered category are randomly distributed or accumulated on top or bottom of the list. Usually, significance scores (p-values) of GSEA are computed by nonparametric permutation tests, a time consuming procedure that yields only estimates of the p-values.

**Results:**

We present a novel dynamic programming algorithm for calculating exact significance values of unweighted Gene Set Enrichment Analyses. Our algorithm avoids typical problems of nonparametric permutation tests, as varying findings in different runs caused by the random sampling procedure. Another advantage of the presented dynamic programming algorithm is its runtime and memory efficiency. To test our algorithm, we applied it not only to simulated data sets, but additionally evaluated expression profiles of squamous cell lung cancer tissue and autologous unaffected tissue.

## Background

Modern high-throughput methods deliver large sets of genes or proteins that can not be evaluated manually. For example, cDNA microarrays are used to measure the expression of a variety of genes under different conditions, e.g. in normal and cancer tissues. Usually, for each gene the expression quotient is computed and the genes are sorted by their expression quotient. The question of interest is whether over-expressed or under-expressed genes accumulate in certain biological categories, as for example biochemical pathways or Gene Ontology categories. To answer this question different approaches can be applied. First, the so-called "Over-Representation Analysis" (ORA) that compares a reference set to a test set of genes by using either the hypergeometric test or Fisher's exact test. Second, "Gene Set Enrichment Analysis" (GSEA) evaluates the distribution of genes belonging to a biological category in a given sorted list of genes or proteins by computing running sum statistics.

**Figure 1:**
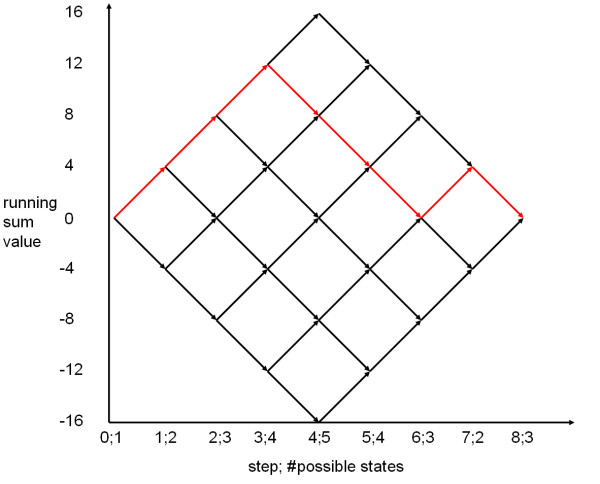
**Example of possible running sum statistics**. The figure shows all possible running sum statistics for an ordered list of 8 genes of which 4 belong to a functional category. The red labeled running sum statistic has a *RS*_*C *_value of 12 and the corresponding p-value is 1−5470=0.229
 MathType@MTEF@5@5@+=feaafiart1ev1aaatCvAUfKttLearuWrP9MDH5MBPbIqV92AaeXatLxBI9gBaebbnrfifHhDYfgasaacH8akY=wiFfYdH8Gipec8Eeeu0xXdbba9frFj0=OqFfea0dXdd9vqai=hGuQ8kuc9pgc9s8qqaq=dirpe0xb9q8qiLsFr0=vr0=vr0dc8meaabaqaciaacaGaaeqabaqabeGadaaakeaacqaIXaqmcqGHsisldaWcaaqaaiabiwda1iabisda0aqaaiabiEda3iabicdaWaaacqGH9aqpcqaIWaamcqGGUaGlcqaIYaGmcqaIYaGmcqaI5aqoaaa@382D@. The numbers on the x-axis refer to the index and the number of possible running sum values in the current step.

Performing GSEA for a biological category *C *and sorted list *L *of *m *genes of which *l *belong to *C *means that a running sum statistic *RS *is computed for *L*. RS statistics evaluate whether the genes of *C *are accumulated on top or bottom of the sorted list or whether they are randomly distributed. Hereby, the sorted list is processed from top to bottom. Whenever a gene belonging to *C *is detected, the running sum is increased by a certain number, otherwise it is decreased. The value of interest is the running sum's maximal deviation from zero, denoted as *RS*_*C*_. An example is provided in Figure 1 for a list containing 8 genes of which 4 belong to *C*. The black graph corresponds to all possible running sum statistics. The red pathway represents the example where the first three genes and the seventh gene belongs to *C*. The *RS*_*C *_value of the red path is 12.

Usually, the p-value is computed by nonparametric permutation tests, i.e. *RS*_*C *_is calculated for permuted gene lists. Two approaches to compute these lists exist. First, the sorted gene list is randomly permuted. Second, if *L *is sorted by the median expression quotient of expression values in one group divided by the median expression value in another group, the samples are randomly assigned to the two groups and thereby permuted gene lists are generated. Notably, these methods do not always yield the same results. The permutation procedure is repeated *t *times and the running sum statistics together with the corresponding maximal deviations from zero, denoted as *RS*_*i*_, *i *∈ {1,...,*t*}, are computed. Usually, the p-value computes as the fraction of *RS*_*i *_values that are larger or equal than *RS*_*C*_:

1t∑i=1tI(RSi≥RSC).
 MathType@MTEF@5@5@+=feaafiart1ev1aaatCvAUfKttLearuWrP9MDH5MBPbIqV92AaeXatLxBI9gBaebbnrfifHhDYfgasaacH8akY=wiFfYdH8Gipec8Eeeu0xXdbba9frFj0=OqFfea0dXdd9vqai=hGuQ8kuc9pgc9s8qqaq=dirpe0xb9q8qiLsFr0=vr0=vr0dc8meaabaqaciaacaGaaeqabaqabeGadaaakeaadaWcaaqaaiabigdaXaqaaiabdsha0baadaaeWbqaaiabdMeajnaabmaabaGaemOuaiLaem4uam1aaSbaaSqaaiabdMgaPbqabaGccqGHLjYScqWGsbGucqWGtbWudaWgaaWcbaGaem4qameabeaaaOGaayjkaiaawMcaaaWcbaGaemyAaKMaeyypa0JaeGymaedabaGaemiDaqhaniabggHiLdGccqGGUaGlaaa@4307@

Since its development in 2003 [1,2], Gene Set Enrichment Analysis has been enhanced [3] and integrated in a number of analysis tools [4]. Among the most popular programs are "ermineJ" [5] and "GSEA-p" [6]. These two tools estimate the significance values by using nonparametric permutation tests. However, such tests entail three disadvantages:

First, repeated runs of the permutation test algorithm may lead to different significance values because of the random sampling.

Second, the permutation test procedure causes problems if the significance values are small. Given a running sum statistic whose true p-value is 0.00001. If, as usual, 1000 permutation tests are performed, probably none will have a higher maximal deviation as the original running sum statistics. According to the formula given above, the p-value would compute as 01000=0
 MathType@MTEF@5@5@+=feaafiart1ev1aaatCvAUfKttLearuWrP9MDH5MBPbIqV92AaeXatLxBI9gBaebbnrfifHhDYfgasaacH8akY=wiFfYdH8Gipec8Eeeu0xXdbba9frFj0=OqFfea0dXdd9vqai=hGuQ8kuc9pgc9s8qqaq=dirpe0xb9q8qiLsFr0=vr0=vr0dc8meaabaqaciaacaGaaeqabaqabeGadaaakeaadaWcaaqaaiabicdaWaqaaiabigdaXiabicdaWiabicdaWiabicdaWaaacqGH9aqpcqaIWaamaaa@3358@, which may be a bad estimation. Since the next iteration may lead to a higher deviation, a more reasonable estimation would be

0≤p-value<1number of permutations.
 MathType@MTEF@5@5@+=feaafiart1ev1aaatCvAUfKttLearuWrP9MDH5MBPbIqV92AaeXatLxBI9gBaebbnrfifHhDYfgasaacH8akY=wiFfYdH8Gipec8Eeeu0xXdbba9frFj0=OqFfea0dXdd9vqai=hGuQ8kuc9pgc9s8qqaq=dirpe0xb9q8qiLsFr0=vr0=vr0dc8meaabaqaciaacaGaaeqabaqabeGadaaakeaacqaIWaamcqGHKjYOcqqGWbaCcqqGTaqlcqqG2bGDcqqGHbqycqqGSbaBcqqG1bqDcqqGLbqzcqGH8aapdaWcaaqaaiabigdaXaqaaiabb6gaUjabbwha1jabb2gaTjabbkgaIjabbwgaLjabbkhaYjabbccaGiabb+gaVjabbAgaMjabbccaGiabbchaWjabbwgaLjabbkhaYjabb2gaTjabbwha1jabbsha0jabbggaHjabbsha0jabbMgaPjabb+gaVjabb6gaUjabbohaZbaacqGGUaGlaaa@5888@

Since GSEA is often applied to many biological categories, p-values have to be adjusted for multiple testing by using Bonferroni Hochberg [7], Benjamini [8], or similar adjustment approaches. However, given the above estimation and the known multiple testing methods, the p-value cannot be adjusted in an appropriate way.

**Figure 2:**
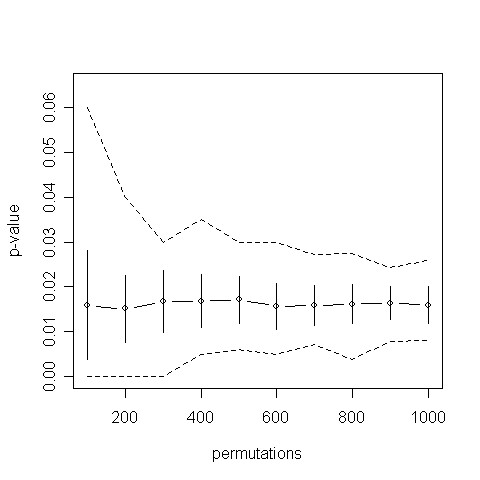
**p-value as function of permutation test number**. For each number of permutation tests we performed 100 runs. The figure shows the mean value of these runs together with the respective standard deviation. The dashed lines represent the maximum and minimum of the 100 computed p-values.

Third, it is difficult to estimate how many permutations should be performed to obtain a sample of reasonable size. Obviously, if *m *= 20000 and *l *= 2000, a sample size of 1000 permutations may be by far too small. Remarkably, the number of possible different running sum statistics amounts to (ml)
 MathType@MTEF@5@5@+=feaafiart1ev1aaatCvAUfKttLearuWrP9MDH5MBPbIqV92AaeXatLxBI9gBaebbnrfifHhDYfgasaacH8akY=wiFfYdH8Gipec8Eeeu0xXdbba9frFj0=OqFfea0dXdd9vqai=hGuQ8kuc9pgc9s8qqaq=dirpe0xb9q8qiLsFr0=vr0=vr0dc8meaabaqaciaacaGaaeqabaqabeGadaaakeaadaqadaqaauaabeqaceaaaeaacqWGTbqBaeaacqWGSbaBaaaacaGLOaGaayzkaaaaaa@3106@. On the example given above, the number of different running sums adds up to approximately 4·10^2821^, emphasizing that 1000 permutation represent a very small sample. The example shown in Figure 2 indicates that for a sorted list of length 2000 and a biological category including only 14 genes 1000 permutations do not yield reliable significance values. The required large number of permutation tests leads to an unacceptable computational effort, especially if thousands of biological categories are tested. An alternative, parametric method is the so called Parametric Analysis of Gene Set Enrichment "PAGE" method [9] that calculates a z-score for a given gene set and infers the significance value of this z-score against standard normal distribution.

In this study, we address the exact and efficient p-value computation for unweighted Gene Set Enrichment Analysis. Unweighted means that the number by which the running sum statistic is increased if a gene of *C *is found and the number by which the running sum statistic is decreased if the gene does not belong to *C *are constants. In our case, whenever a gene of *C *is found the running sum is increased by *m *- *l*, and otherwise it is decreased by *l*. The dynamic programming method is similar to the "DRIM" approach ([10]) that computes the optimal partition of a gene set in a target and a background set.

We integrated our dynamic programing algorithm into the gene set analysis tool "GeneTrail" [11] that is freely available at *genetrail.bioinf.uni-sb.de*. GeneTrail tests a wide variety of biological categories, among them Kyoto Encyclopedia of Genes and Genomes (KEGG) pathways [12], TRANSPATH pathways [13], transcription factors [14], Gene Ontology GO, [15], granzyme B clevage sites [16], and protein-protein interactions [17-20]. GeneTrail relies on the Biological Information System BN++ [21] that provides easy access to a wide variety of biological data.

## Results and Discussion

### Dynamic programming algorithm

Before presenting our algorithm, we discuss some important features of the running sum statistic. Given the sorted list *L *of *m *genes of which *l *belong to the considered biological category *C*, we calculate a running sum statistic as follows: whenever we find one of the *l *genes of the considered category *C*, we increase the running sum by *m *- *l *leading to a total sum of *l*·(*m *- *l*) over all genes in *C*. Otherwise, we decrease the running sum statistic by *l *leading to a total sum of (*m *- *l*)·(-*l*) over all genes not in *C*. Therefore, the running sum's final value will always be zero. Moreover, the running sum's maximal possible value is *l*(*m *- *l*), whereas its minimal possible value is -*l*(*m *- *l*).

As mentioned in the Introduction, the value of interest is the running sum's maximal deviation from zero, denoted as *RS*_*C*_. The p-value can be computed as the probability that a random running sum reaches a maximal deviation greater or equal as *RS*_*C*_. We compute this probability via the complement of the event as:

1−XY,
 MathType@MTEF@5@5@+=feaafiart1ev1aaatCvAUfKttLearuWrP9MDH5MBPbIqV92AaeXatLxBI9gBaebbnrfifHhDYfgasaacH8akY=wiFfYdH8Gipec8Eeeu0xXdbba9frFj0=OqFfea0dXdd9vqai=hGuQ8kuc9pgc9s8qqaq=dirpe0xb9q8qiLsFr0=vr0=vr0dc8meaabaqaciaacaGaaeqabaqabeGadaaakeaacqaIXaqmcqGHsisldaWcaaqaaiabdIfaybqaaiabdMfazbaacqGGSaalaaa@31ED@

where *X *is the number of running sum statistics with a maximum deviation of at most *RS*_*C *_- 1 and *Y *is the number of all possible different running sum statistics which can be obviously computed as (ml)
 MathType@MTEF@5@5@+=feaafiart1ev1aaatCvAUfKttLearuWrP9MDH5MBPbIqV92AaeXatLxBI9gBaebbnrfifHhDYfgasaacH8akY=wiFfYdH8Gipec8Eeeu0xXdbba9frFj0=OqFfea0dXdd9vqai=hGuQ8kuc9pgc9s8qqaq=dirpe0xb9q8qiLsFr0=vr0=vr0dc8meaabaqaciaacaGaaeqabaqabeGadaaakeaadaqadaqaauaabeqaceaaaeaacqWGTbqBaeaacqWGSbaBaaaacaGLOaGaayzkaaaaaa@3106@. To compute *X*, we count all running sum statistics that have a maximum deviation of at most *RS*_*C *_- 1.

We use a matrix *M *of dimension (2*l*(*m *- *l*) + 1) × (*m *+ 1), where the different rows represent all possible values of the running sum and the columns represent the indices of the sorted list *L *from 1,..., *m *and an initialization column with index 0. Let *M*(*j*, *i*) denote the number of running sum statistics with value *j *in step *i *whose maximum deviation of zero is less than *RS*_*C *_- 1. The entries of *M *are computed using dynamic programming, starting with the first column. *M*(0, 0) is set to 1 and all other values are set to 0.

We fill the matrix column by column, where the matrix entry *M*(*j*, *i*) is recursively computed as:

M(j,i)={M(j−m+l,i−1)+M(j+l,i−1)if (∗)0else
 MathType@MTEF@5@5@+=feaafiart1ev1aaatCvAUfKttLearuWrP9MDH5MBPbIqV92AaeXatLxBI9gBaebbnrfifHhDYfgasaacH8akY=wiFfYdH8Gipec8Eeeu0xXdbba9frFj0=OqFfea0dXdd9vqai=hGuQ8kuc9pgc9s8qqaq=dirpe0xb9q8qiLsFr0=vr0=vr0dc8meaabaqaciaacaGaaeqabaqabeGadaaakeaacqWGnbqtcqGGOaakcqWGQbGAcqGGSaalcqWGPbqAcqGGPaqkcqGH9aqpdaGabeqaauaabeqaciaaaeaacqWGnbqtcqGGOaakcqWGQbGAcqGHsislcqWGTbqBcqGHRaWkcqWGSbaBcqGGSaalcqWGPbqAcqGHsislcqaIXaqmcqGGPaqkcqGHRaWkcqWGnbqtcqGGOaakcqWGQbGAcqGHRaWkcqWGSbaBcqGGSaalcqWGPbqAcqGHsislcqaIXaqmcqGGPaqkaeaacqqGPbqAcqqGMbGzcqqGGaaicqGGOaakcqGHxiIkcqGGPaqkaeaacqaIWaamaeaacqqGLbqzcqqGSbaBcqqGZbWCcqqGLbqzaaaacaGL7baaaaa@5A06@

where the constraint

(*) -|*RS*_*C*_| <*j *< |*RS*_*C*_|

ensures that only the running sum statistics with maximal deviation of smaller than *RS*_*C *_are counted. The total number of running sum statistics with maximum deviation smaller than *RS*_*C *_can be found at matrix entry *M*(0, *m*).

### Implementation details

At first glance, the presented algorithm seems to be inefficient concerning both, space requirement and runtime, which are of order *O*(*m*^2^*l*). For example, if *m *= 20000 genes and a functional category with *l *= 2000 genes is considered, *M *would have about 1.44·10^12^entries.

We have implemented the above described algorithm in C++ using time and space efficient data structures which will be discussed here.

As the recurrence equation implies, filling the *i*th column of *M *only requires the values of the *i *- 1th column. Thus, the dynamic programming approach requires only two columns of the matrix reducing the memory requirements to *O*(*ml*).

**Figure 3:**
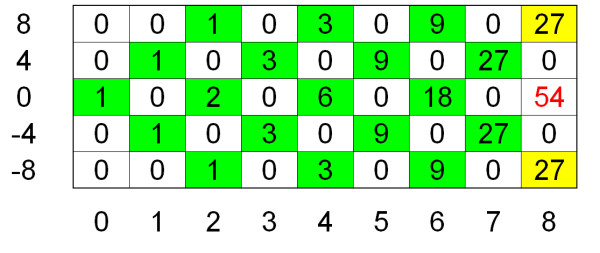
**Dynamic Programming Matrix**. The figure shows the dynamic programming matrix for the example provided in Figure 1. Matrix entries that are unequal zero are shaded. The yellow matrix entries do not have to be computed due to the extended side constraints, the number of running sum statistics with a smaller deviation of zero (*RS*_*C *_value) than 12 amounts to 54.

Obviously, the first column *M*(·, 0) contains only one number unequal to zero, the second column two numbers, and the *l*th column *l *values unequal to zero (see Figure 1 and Figure 3). By using two standard STL hash maps instead of the matrix *M*, the space requirements can be reduced to *O*(*l*) and the expected runtime can be reduced to *O*(*ml*).

Another important feature of the running sum statistics implies that certain parts of the matrix *M *do not have to be computed. The running time of the algorithm can be further reduced by adding a second constraint

(**) -*m*^2 ^+ *l*·*m *+ *i*·*m *- *i*·*l *≤ *j *≤ *l*·*m *- *i*·*l*

for each column *i *to the recurrence equation. The right side of the constraint holds because, for column *i*, the value *j *of the running sum can be computed as

*j *= *a*·(*m *- *l*) + (*i *- *a*)·(-*l*)

where *a *is the number of genes that belong to *C *up to index *i *in the ordered list. Since *a *can be at most *l*, the following inequality holds

j⇔j⇔j ≤≤≤ l⋅(m−l)+(i−l)⋅(−l)l⋅m−i⋅ll⋅(m−i)
 MathType@MTEF@5@5@+=feaafiart1ev1aaatCvAUfKttLearuWrP9MDH5MBPbIqV92AaeXatLxBI9gBaebbnrfifHhDYfgasaacH8akY=wiFfYdH8Gipec8Eeeu0xXdbba9frFj0=OqFfea0dXdd9vqai=hGuQ8kuc9pgc9s8qqaq=dirpe0xb9q8qiLsFr0=vr0=vr0dc8meaabaqaciaacaGaaeqabaqabeGadaaakeaafaqaceWabaaabaGaemOAaOgabaGaeyi1HSTaemOAaOgabaGaeyi1HSTaemOAaOgaaiabbccaGuaabeqadeaaaeaacqGHKjYOaeaacqGHKjYOaeaacqGHKjYOaaGaeeiiaasbaeaabmqaaaqaaiabdYgaSjabgwSixlabcIcaOiabd2gaTjabgkHiTiabdYgaSjabcMcaPiabgUcaRiabcIcaOiabdMgaPjabgkHiTiabdYgaSjabcMcaPiabgwSixlabcIcaOiabgkHiTiabdYgaSjabcMcaPaqaaiabdYgaSjabgwSixlabd2gaTjabgkHiTiabdMgaPjabgwSixlabdYgaSbqaaiabdYgaSjabgwSixlabcIcaOiabd2gaTjabgkHiTiabdMgaPjabcMcaPaaaaaa@65F3@

Equivalently, for the left side of constraint (**) and column *i *the following equation holds:

j⇔j == (i−b)⋅m−i⋅l−b⋅m+i⋅m−i⋅l
 MathType@MTEF@5@5@+=feaafiart1ev1aaatCvAUfKttLearuWrP9MDH5MBPbIqV92AaeXatLxBI9gBaebbnrfifHhDYfgasaacH8akY=wiFfYdH8Gipec8Eeeu0xXdbba9frFj0=OqFfea0dXdd9vqai=hGuQ8kuc9pgc9s8qqaq=dirpe0xb9q8qiLsFr0=vr0=vr0dc8meaabaqaciaacaGaaeqabaqabeGadaaakeaafaqaceGabaaabaGaemOAaOgabaGaeyi1HSTaemOAaOgaaiabbccaGuaabeqaceaaaeaacqGH9aqpaeaacqGH9aqpaaGaeeiiaasbaeaabiqaaaqaaiabcIcaOiabdMgaPjabgkHiTiabdkgaIjabcMcaPiabgwSixlabd2gaTjabgkHiTiabdMgaPjabgwSixlabdYgaSbqaaiabgkHiTiabdkgaIjabgwSixlabd2gaTjabgUcaRiabdMgaPjabgwSixlabd2gaTjabgkHiTiabdMgaPjabgwSixlabdYgaSbaaaaa@562E@

where *b *is the number of genes that do not belong to *C *up to index *i *in the ordered list. Since *b *can be at most *m *- *l*,

j⇔j ≥≥ −(m−l)⋅m+i⋅m−i⋅l−m2+m⋅l+i⋅m−i⋅l
 MathType@MTEF@5@5@+=feaafiart1ev1aaatCvAUfKttLearuWrP9MDH5MBPbIqV92AaeXatLxBI9gBaebbnrfifHhDYfgasaacH8akY=wiFfYdH8Gipec8Eeeu0xXdbba9frFj0=OqFfea0dXdd9vqai=hGuQ8kuc9pgc9s8qqaq=dirpe0xb9q8qiLsFr0=vr0=vr0dc8meaabaqaciaacaGaaeqabaqabeGadaaakeaafaqaceGabaaabaGaemOAaOgabaGaeyi1HSTaemOAaOgaaiabbccaGuaabeqaceaaaeaacqGHLjYSaeaacqGHLjYSaaGaeeiiaasbaeaabiqaaaqaaiabgkHiTiabcIcaOiabd2gaTjabgkHiTiabdYgaSjabcMcaPiabgwSixlabd2gaTjabgUcaRiabdMgaPjabgwSixlabd2gaTjabgkHiTiabdMgaPjabgwSixlabdYgaSbqaaiabgkHiTiabd2gaTnaaCaaaleqabaGaeGOmaidaaOGaey4kaSIaemyBa0MaeyyXICTaemiBaWMaey4kaSIaemyAaKMaeyyXICTaemyBa0MaeyOeI0IaemyAaKMaeyyXICTaemiBaWgaaaaa@6223@

Although the additional constraint does not lead to an asymptotically improved runtime, an increased performance has been measured, especially for small p-values.

Additionally, the runtime of the presented algorithm can be improved by computing only the first half of *M*. Due to a certain "symmetry" of the running sum statistics it suffices to compute either the column in the middle or the two columns in the middle to derive the required number of pathways.

### Dependence of runtime on p-value

**Figure 4:**
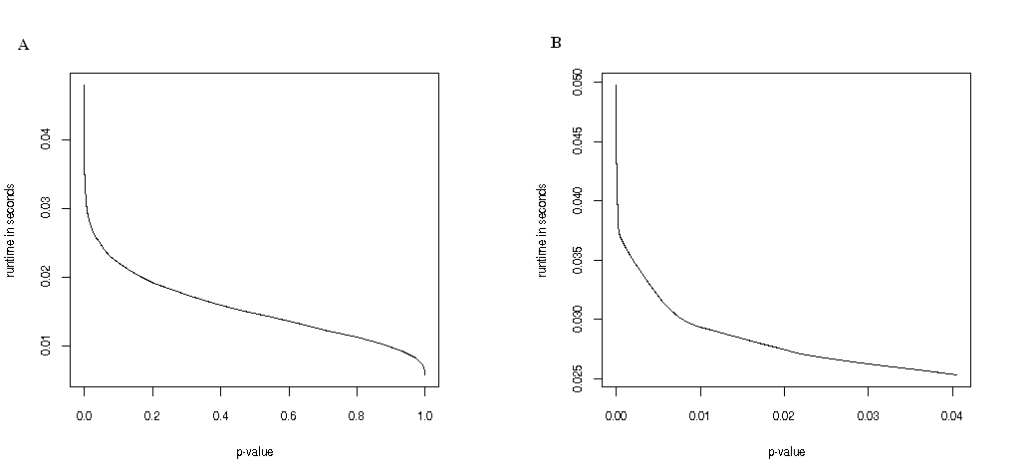
Running time as function of the significance value. A: For a sorted list of 1000 genes and a category containing 100 genes the runtime of our algorithm was computed for a set of discrete p-values (0, 0.003, 0.006,...,0.996, 1). The shown runtime is the median of 100 runs for each p-value on a standard 2 GHz PC. The maximal runtime was 0.05 seconds spent for computing the complete matrix. A naive permutation test procedure needed about 0.7 seconds for 1000 permutations. B: Running time as function of the significance value for small p-values.

The recurrence equation of the dynamic programming algorithm shows that in each step only computations for running sum values in the interval of ] - |*RS*_*C*_|, |*RS*_*C*_|[ have to be computed. However, the running sum's maximal deviations from zero and the corresponding p-values are mutually dependent on each other, i.e., the higher the running sum's maximal deviation, the lower the p-value. Thus, the running time of the presented algorithm depends on the p-value (see Figure 4A and 4B). The lower the p-values are the higher is the runtime of the presented algorithm.

As described above, our algorithm is applied to evaluate several thousands of biological categories/hypotheses using the gene set analysis toolkit "GeneTrail". In general, findings are considered to be significant, if the p-value is smaller than 0.05. Most computation time is spend for small p-values. However, only few of the considered categories are statistically significant, whereas the others will lead to intermediate and larger p-values. Since our algorithm is especially fast for intermediate and large p-values, the complete GSEA analysis is highly efficient and most of its running time is spent for the p-value calculation of the most significant categories.

### Comparison to other approaches

To get maximal performance, we implemented our algorithm in C++. Other available GSEA tools have been implemented in Java or are available as "R" scripts. For this reason, a fair comparison of our tool to other nonparametric permutation tests is not possible. We implemented a permutation test procedure in C++ with expected running time of *O*(number of permutations·m). We applied the algorithm to the example presented in Figure 4 using 1000 permutations. On average the presented dynamic programming approach was more than ten times faster compared to the permutation test procedure. Please note that the two approaches are not directly comparable. The runtime of both methods depends on the length of the gene list *l*. In addition, the runtime of our algorithm depends on the p-value whereas the running time of the permutation test approach depends on the number of performed permutations.

### Evaluation of lung cancer expression profiles

We tested our algorithm by evaluating freely available expression profiles of lung cancer tissue and autologous control samples. In detail, we downloaded expression profiles of 5 squamous cell lung cancer patients [22] from the "Gene Expression Omnibus" [23]. Together with the cancer tissue, unaffected tissue of autologous patients was extracted at surgery and 5 control expression profiles were generated. The 10 expression profiles were measured using the Affymetrix HG-U133A including more than 22000 transcripts and 13000 genes. In a pre-processing step, the profiles were median normalized. Thereafter, for each transcript a paired t-tests was performed in order to detect differentially expressed genes. Paired t-test is applicable here, since the control samples were taken from the normal lung tissues of autologous patients. To generate a sorted list, t-test statistic values were sorted in increasing order such that the top of the resulting list contains the most significantly up-regulated genes in lung-cancer and the bottom of the list the most significantly down-regulated genes.

Finally, the list was analyzed by GeneTrail. A detailed evaluation of all findings would be beyond the scope of this paper. Therefor, we provide a summary of our findings in Table 1 and the complete list of results in the supplemental material. Some significant findings pointing out the advantages of the presented dynamic programming algorithm are given below. Although the evaluation of the data set revealed a variety of significant categories (see Table 1), the complete analysis needed only about one hour. Of 6428 categories, 1744 were statistically significant at an *α*-level of 0.05 without adjustment, and 711 with Benjamini-Hochberg adjustment. About 300 categories would achieve a p-value of zero by application of 1000 permutation tests. However, these categories differ extremely in their significance.

**Table 1:** Evaluation on lung cancer

**Category**	**#sub-categories**	**#significant**	**#significant adjusted**
KEGG	165	44	22
TRANSPATH	171	28	4
TRANSFAC	248	41	5
Gene Ontology	5771	1590	646
Chromosomes	25	15	11
Chromosome Arms	44	24	23
Sequence Motifs	4	2	2
Sum	6428	1744	711

We detected many significantly down-regulated KEGG-pathways. Among them the *Cell Adhesion Molecules *(p-value of 0.00024). The *Cell Cycle *is the most significantly up-regulated pathway (p-value of 0.0011). It is very likely that both pathways would achieve a p-value of zero by permutation tests, however, they are not equally significant as demonstrated above. The up-regulated *rRNA-binding *achieved a p-value of 0.0488. This category represents an example where permutation tests might define a pathway as significant in one run and as not significant in another run.

## Conclusion

We presented a novel dynamic programming algorithm that enables the efficient computation of exact significance values of unweighted "Gene Set Enrichment Analysis" and thus avoids typical problems of nonparametric permutation tests. Additionally, we showed that the runtime of the presented algorithm decreases as the p-values increase, i.e. our algorithm spends most time for computing small p-values of significant categories.

We integrated our algorithm in the gene set analysis tool "GeneTrail" that allows for performing a wide variety of statistical analyses efficiently. Using GeneTrail, we evaluated the differential expression of genes in squamous cell lung cancer expression profiles, demonstrating the usefulness of the presented dynamic programming algorithm.

## Methods

### GMP library for arbitrary numbers

The number of possible running sum statistics increases exponentially, i.e. (ml)
 MathType@MTEF@5@5@+=feaafiart1ev1aaatCvAUfKttLearuWrP9MDH5MBPbIqV92AaeXatLxBI9gBaebbnrfifHhDYfgasaacH8akY=wiFfYdH8Gipec8Eeeu0xXdbba9frFj0=OqFfea0dXdd9vqai=hGuQ8kuc9pgc9s8qqaq=dirpe0xb9q8qiLsFr0=vr0=vr0dc8meaabaqaciaacaGaaeqabaqabeGadaaakeaadaqadaqaauaabeqaceaaaeaacqWGTbqBaeaacqWGSbaBaaaacaGLOaGaayzkaaaaaa@3106@. On the example given above, a microarray containing *m *= 20000 genes and a category with *l *= 2000 genes, the number of different running sums adds up to approximately 4·10^2821^. In the worst case, the matrix entry *M*(0, *m*) amounts to 4·10^2821^, if all genes of *C *are either top or bottom ranked. This example shows that the approach must be able to handle very large numbers. Hence, we use the "GNU Multiple Precision Arithmetic Library" (GMP), a numerically stable and fast library that can compute arbitrary large natural numbers and is freely available.

## Authors' contributions

AK developed and implemented the dynamic programming algorithm and wrote the manuscript. CB integrated the algorithm in GeneTrail and supported the implementation of the algorithm. HPL is the senior author of the study.
